# Genome-wide CRISPR screen identifies ESPL1 limits the response of gastric cancer cells to apatinib

**DOI:** 10.1186/s12935-024-03233-4

**Published:** 2024-02-24

**Authors:** Bei Zhang, Yan Chen, Xinqi Chen, Zhiyao Ren, Hong Xiang, Lipeng Mao, Guodong Zhu

**Affiliations:** 1 Institute of Gerontology, Guangzhou Geriatric Hospital, Guangzhou Medical University, Guangzhou, China; 2 State Key Laboratory of Respiratory Disease, Guangzhou Geriatric Hospital, Guangzhou Medical University, Guangzhou, China; 3 Collaborative Innovation Center for Civil Affairs of Guangzhou, Guangzhou, China; 4 Department of Geriatrics, The First Affiliated Hospital of Chongqing Medical University, Chongqing, China; 5 Department of Oncology, Affiliated Dongguan Hospital, Southern Medical University, Dongguan, China; 6 Departments of Oncology, School of Medicine, Guangzhou First People’s Hospital, South China University of Technology, Guangzhou, China; 7 Department of Systems Biomedical Sciences, School of Medicine, Jinan University, Guangzhou, China

**Keywords:** Apatinib resistance, ESPL1, CRISPR screening, MDM2, Gastric cancer

## Abstract

Apatinib was the first anti-angiogenic agent approved for treatment of metastatic gastric cancer (GC). However, the emergence of resistance was inevitable. Thus investigating new and valuable off-target effect of apatinib directly against cancer cells is of great significance. Here, we identified extra spindle pole bodies-like 1 (ESPL1) was responsible for apatinib resistance in GC cells through CRISPR genome-wide gain-of-function screening. Loss of function studies further showed that ESPL1 inhibition suppressed cell proliferation, migration and promoted apoptosis in vitro, and accordingly ESPL1 knockdown sensitized GC cells to apatinib. In addition, we found ESPL1 interacted with mouse double minute 2 (MDM2), a E3 ubiquitin protein ligase, and the combination of MDM2 siRNA with apatinib synergistically ameliorated the resistance induced by ESPL1 overexpression. In summary, our study indicated that ESPL1 played a critical role in apatinib resistance in GC cells. Inhibition of MDM2 could rescue the sensitivity of GC cells to apatinib and reverse ESPL1-mediated resistance.

**Supplementary Information:**

The online version contains supplementary material available at 10.1186/s12935-024-03233-4.

## Introduction

According to the GLOBOCAN 2020, gastric cancer (GC) is the fifth most commonly diagnosed cancer worldwide and the fourth leading cause of cancer-related death, with approximately 1,089,103 new cases and 768,793 deaths in 2020 [1]. Although several molecular targets have been investigated for GC, the prognosis for the metastatic GC remains poor with median survival less than 1 year [2]. Apatinib, a highly selective inhibitor of vascular endothelial growth factor receptor 2, has been approved and indicated for advanced GC after the failure of two or more lines of systemic therapy in China [3]. Previous studies have shown that low-dose apatinib could optimizes tumor microenvironment and potentiate antitumor effect of PD-1/PD-L1 blockade in lung cancer [4], and significantly improved the overall survival in patients with pretreated advanced hepatocellular carcinoma compared with placebo [5]. However, the emergence of resistance is inevitable. Thus, investigating new and valuable off-target effect of apatinib directly against cancer cells, and finding molecular targets for combined therapy is of great clinical significance to improve the prognosis of patients with advanced GC.

Extra spindle pole bodies-like 1 (ESPL1) was a caspase-like protease, which played a central role in cell cycle progression to ensure immaculate genetic inheritance [6]. Besides its canonical role, ESPL1 was required for centrosome duplication, DNA damage repair and so on. Both premature and delayed activation of ESPL1 would result in chromosomal instability, which was controlled by binding to different partners securin, CDK1-cyclin B or MAD2-SGO2 [6, 7]. It was reported that ESPL1 was overexpressed in different malignant tumors, such as breast cancer and glioma [8, 9] and ESPL1 was shown to have oncogenic activity in mouse model [10]. In addition, ESPL1 played a central protective role in cancer cells by converting the normally protective proteins MCL1 and Bcl-XL into pro-apoptotic agents [11]. However, whether ESPL1 was involved in apatinib resistance has not yet been studied.

Currently, we found several new targets MCM2, CCND3, ESPL1 and PLK1 responsible for apatinib resistance by CRSIPR gain-of-function screening. ESPL1 inhibition could enhance the sensitivity of GC cells to apatinib. In addition, down-regulation of mouse double minute 2 (MDM2) could rescue the sensitivity of GC cells to apatinib and reverse ESPL1-mediated resistance. In summary, our results demonstrated that targeting ESPL1 might be an effective approach to overcome apatinib resistance in GC.

## Materials and methods

### Cell lines

Human gastric cancer cells AGS, HGC27, BGC823 and NCI-N87 was cultured in RPMI 1640 medium (HyClone), with 10% fetal bovine serum (FBS, Gibco) and 0.1% Penicillin/Streptomycin (P/S, TBD, PS2004HY), obtained from Procell (Wu Han, China). The cells were placed in a humidified incubator with 5% CO2 at 37 °C.

### Genome-wide CRISPR/Cas9 activation screen

The genome wide CRISPR/Cas9 SAM lentiviral library (Addgene plasmid #1,000,000,078), containing 70,290 unique sgRNA sequences targeting 23,430 human genes**,** was introduced into AGS cells by lentiviral transduction as previously described [12]. For SAM gain-of-function screening, this gRNA library must be combined with 1 additional SAM construct MS2-P65-HSF1 (Addgene plasmid #89,308), selected with hygromycin B (YEASEN, 60225ES03). The AGS cells used for pooled SAM human lentiviral library transduction should stably expressing MS2-P65-HSF1. Then, at least 1 × 10^8^ AGS cells were transduced with pooled SAM lentiviral library at a low MOI (~ 0.5). Lastly, the transduced cells were selected with blasticidin (MDBio, D0120601) for 10 days to generate a mutant cell pool. Cells stably expressing SAM gRNAs library were cultured with 10 μg/mL apatinib (Hengrui Pharmaceutical Co., Ltd.) for two rounds. Before starting the next round of treatment, cells were allowed to grow to fusion and resistant cells were collected for genomic DNA extraction.

### Genomic DNA extraction and sgRNA deep sequencing

The HiPure Tissue DNA Mini Kits (Magen) were used to extract genomic DNA. Amplification of the sgRNA sequences of each sample from the extracted genomic DNA using the CRISPRa-F: TCTTGTGGAAAGGACGAAACACCG and CRISPRa-R: CTCCTTTCAAGACCTAGGATC primers [12, 13]. The pooled PCR products were gel purified using the QiaQuick kit (Qiagen). The sgRNA abundance in the pooled resistant cells was detected by Illumina deep sequencing (Novogene Technology, China).

### Establishment of candidate genes overexpression GC cells

The candidate genes MCM2, CCND3, ESPL1, PLK1, TCF7, PRICKLE1 and VANGL1 were selected for validation.The sgRNA plasmids of the candidate genes were synthesized(GENEROL BIOL) and used for GC cells transfection. Lentivirus was produced in 293 T cells and 9 µg sgRNA plasmid, 3 µg pCMV-VSVG plasmid, 3 µg pMDLg pRRE plasmid and 3 µg pRSV-Rev plasmid were used. The AGS and HGC27 cells were transfected with the sgRNA lentivirus. Then, the cells were selected with blasticidin (MDBio, D0120601).

### shRNA and siRNA transfection

AGS or HGC27 cells were seeded in 6-well plates and transient transfection with ESPL1 shRNA (Table 1) or MDM2 siRNA (Table 2) when the cells reached 60% confluency. Briefly, 6μL shRNA or siRNA was pre-mixed with 100μL Opti-MEM (Gibco). Then, 8µL Lipo8000 transfection reagent (Beyotime, C0533) was added into 100 Opti-MEM and dropped into the plasmid mixture. The complex were incubated for 20 min at room temperature and then added to AGS or HGC27 cell culture. Transfection media was removed 5 h later and replaced with RPMI 1640 complete medium. The shRNA and siRNA were obtained from GENERAL BIOL (Chuzhou, China).

**Table 1:** The shRNA sequences

Primer name	Sequences
ESPL1-shRNA-a	GCTGCTGTACTACCCAACTTT
ESPL1-shRNA-b	CCGCTTCTTACACCAGTAATT

**Table 2:** The siRNA targeting sequences

Primer name	Sequences(5'to3')
MDM2 siRNA-a	UGAAGAAGAUCCUGAAAUUTT
	AAUUUCAGGAUCUUCUUCATT
MDM2 siRNA-b	GCUUCACAAUCACAAGAAATT
	UUUCUUGUGAUUGUGAAGCTT
MDM2 siRNA-c	GGAUCUUGAUGCUGGUGUATT
	UACACCAGCAUCAAGAUCCTT

### Real-time quantitative reverse transcriptase PCR

The MCM2, CCND3, ESPL1, PLK1, TCF7, PRICKLE1 and VANGL1 mRNA levels were detected by real-time quantitative reverse transcriptase PCR (q-PCR) as previously described [12]. The PCR primers used were shown in Table 3.

**Table 3:** Primers sequences for q-PCR

Gene symbol	Accession number	Product size	Primer sequences
β-actin	HQ154074	185 bp	F: TGGCACCAGCACAATGAA
			R: CTAAGTCATAGTCCGCCTAGAAGCA
MCM2	NM_004526	162 bp	F: GCCATGCCCAACACGTATG
			R: GCCTGTCGCCATAGATTCTTTC
CCND3	NM_001136017	239 bp	F: GCCCCTGACCATCGAAAAAC
			R: TGGCAAAGGTATAATCTGTAGCAC
PLK1	NM_005030	117 bp	F: GTACGGCCTTGGGTATCAGC
			R: GTGCCGTCACGCTCTATGTA
TCF7	NM_001134851	182 bp	F: GCTGCCATCAACCAGATCCT
			R: GTGGATTCTTGGTGCTTTTCCC
PRICKLE1	NM_001144881	189 bp	F: TGAGTGCACAGAAGCTGAGG
			R: TGTGCATGGTCCACACCAAT
VANGL1	NM_138959	123 bp	F: AGCCTGGGACACCTGAGTATC
			R: CATATGCTTGGCTGCTCGGA
ESPL1	NM_012291	225 bp	F: TCAAAGAGTATGGGGCCTCG
			R: CCCCATGCCCTGCATAGATA

### Cell proliferation assay

The effect of apatinib (10 μg/mL, 48 h) on cells transfected with MCM2, CCND3, ESPL1, PLK1, TCF7, PRICKLE1 and VANGL1 overexpression plasmids was detected by CCK-8 assays (Beyotime, C0039) according to the manufacturer’s instructions. AGS-NC, AGS-ESPL1, AGS-shNC, AGS-shESPL1 cells were seeded in 96-well plates and treated with apatinib at the concentration of 0, 1, 10, 40 μg/mL for 48 h before running assay. Cell growth inhibition rate was then calculated based on the absorbance.

### Migration Assay

The transwell (Corning) migration assay was used to detect the effect of apatinib (10 μg/mL, 48 h) on cells transfected with MCM2, CCND3, ESPL1, PLK1, TCF7, PRICKLE1 and VANGL1 overexpression plasmids as previously described [13].

### Apoptosis assay

Cell apoptosis was conducted after treatment with10μg/mL apatinib or DMSO vehicle for 48 h using APC-conjugated Annexin V (Annexin V-APC) and 7-aminoactinomycin D (7-AAD) (Multisciences, AP104-100) according to manufacturers’ protocols. Fluorescence was measured using a BD FACS Calibur flow cytometer (Beckman) and data were analyzed by FlowJo software (FlowJo).

### Western Blot analysis

The expression of ESPL1 (Santa, sc-390314), MDM2 (Santa, sc-965), Phospho-pan-AKT1/2/3 (affbiotech, AF0016), VEGF (HUABIO, ET1604-28), BCL-2 (Proteintech, 12,789–1-AP) protein was detected by western blot as previously described [13]. GAPDH (Proteintech, HRP-60004) was used as an internal reference.

### Co-immunoprecipitation assay

Co-Immunoprecipitation (Co-IP) was used to detect the protein interactions. Firstly, the AGS cells are prepared and lysated with 200ul RIPA buffer (Beyotime, P0013B). Protein A/G MagBeads (GenScript, L00277) were incubated with ESPL1 (Santa, sc-390314) and MDM2 (Santa, sc-965) antibodies overnight, respectively. Then the MagBeads-antibody complexes were washed with 500ul IP Buffer for 3 times and incubated with cell lysates overnight to capture its direct interacting partners. Finally, MagBeads–antibody–protein complexes are precipitated and analyzed by Western Blot.

### TCGA data analysis

For gastric cancer (Stomach adenocarcinoma, STAD) samples in TCGA, mRNA seq were retrieved and convert FPKM data to TPM. Finally, the mRNA expression level of MCM2, CCND3, ESPL1, PLK1, TCF7, PRICKLE1 and VANGL1 in 380 cancers and 37 paracancerous samples were analysized.

### Drug susceptibility analysis

RNA-sequencing expression profiles and corresponding clinical information for STAD were downloaded from the TCGA dataset. The samples were devided into low and high expression group according to the median of ESPL1 mRNA levels. The chemotherapeutic response for each sample was predicted by R package “pRRophetic” based on the Genomics of Drug Sensitivity in Cancer (GDSC, https://www.cancerrxgene.org). The samples' half-maximal inhibitory concentration (IC_50_) was estimated by ridge regression. All parameters were set as the default values. Using the batch effect of combat and tissue type of all tissues, and the duplicate gene expression was summarized as mean value.

### sgRNA-Seq data analysis

After the resistant cells was pooled to detect the sgRNA abundance by Illumina deep sequencing. The R software package (DESeq2) was then applied to perform a statistical analysis of the sequencing data as was shown in Additional file 2: Table **S1**. KEGG pathway enrichment analysis for DEGs were performed with R package topGO (v2.44.0). KEGG pathway with *P* value < 0.05 were considered as significantly enriched. The normalized expression matrix from DESeq2 was further centered and scaled by scale function and then visualized by R package pheatmap (v1.0.12).

## Results

### CRISPR activation screening for apatinib resistance

**Fig. 1:**
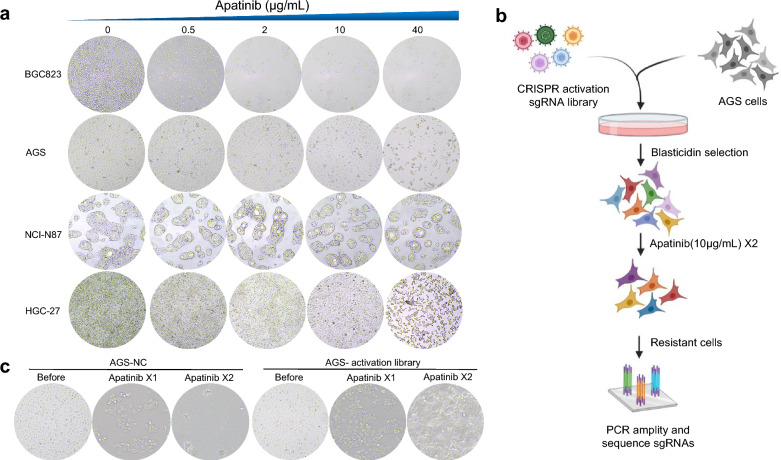
Schematic of functional screening by CRISPR/Cas9 SAM pooled library and apatinib treatment. (a) Gastric cancer cell lines: AGS, HGC-27, NCI-N87 and BGC823 were treated with different concentrations of apatinib (0, 0.5, 2, 10, 40 μg/mL) for 24 h and the cell viability was observed; b) Schematic of apatinib-resistant GC cells construction for high-throughput sequencing analysis; c) Optical microscopic images of GC cells transfected with lentiviral sgRNA library and treated with apatinib for two rounds

In this study, the human CRISPR activation pooled library was used to identify genes responsible for apatinib resistance in GC cells. The GC cell lines (AGS, HGC-27, NCI-N87 and BGC823) were treated with different concentrations of apatinib (0, 0.5, 2, 10, 40 μg/mL) for 24 h and the viability of GC cells was observed. The results showed that the GC cells died obviously after apatinib treatment for 24 h. In particular, nearly all BGC823 cells, 70% AGS cells and 50% HGC27 cells were died when treated with 10 μg/mL (Fig. 1a). Figure 1b displayed the schematic of apatinib resistant AGS cells enrichment for sgRNA sequencing. In detail, the mutant cell pool (at least 5 × 10^7^ AGS cells) was treated with 10 μg/mL apatinib for two rounds (7 days per round), then the survival cells were enriched (Fig. 1c) and collected to extract DNA. PCR amplification of the 209-bp sgRNA region was used for high-throughput sequencing to calculate the sgRNAs coverage in the resistant AGS cells.

### Candidate genes were selected in apatinib-resistant cells

**Fig. 2:**
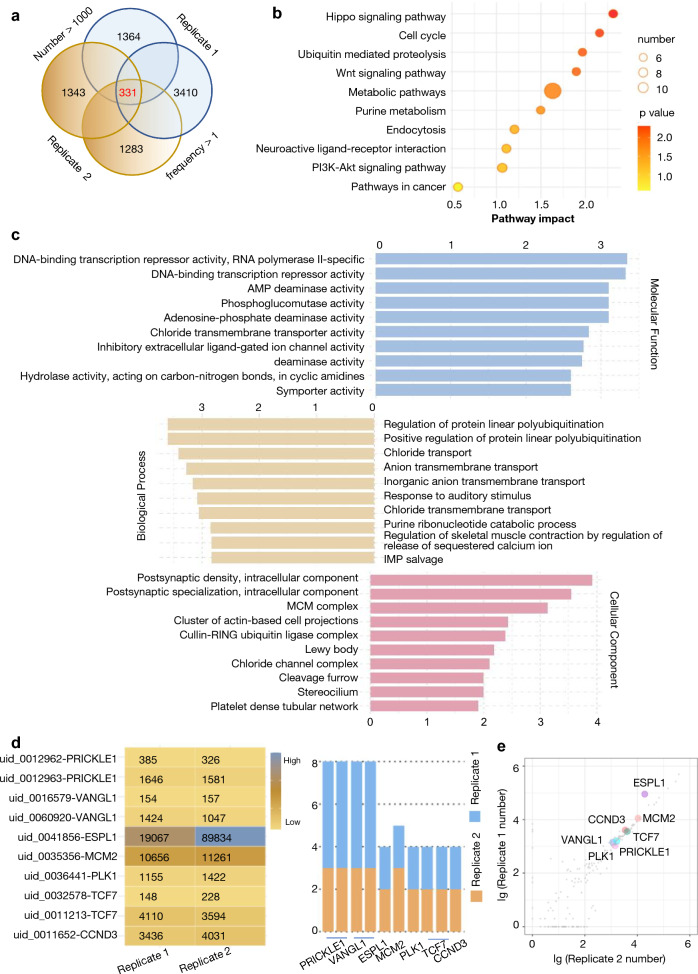
Enriched apatinib-resistant genes from the CRISPR/Cas9 activation screen in GC. **a** The Venn diagram showed 332 genes with sgRNA abundance greater than 1000 and sgRNA frequency greater than 1 in two replicate samples were selected for further analyses; **b** The top 10 enriched pathways by KEGG analysis; **c** The top 10 terms of molecular function, biological process and cellular component enriched in GO enrichment analysis; **d** The heatmap and bar graph showed the counts of resistant sgRNAs (left) and the sgRNA frequency (right) of the selected 7 genes; **e** Scatter plot showed sgRNAs targeting the intersected 332 genes in two replicate samples and the 7 candidate genes were marked

The enriched resistant AGS cells were used for sgRNA sequence, a total of 15,312 and 8274 sgRNAs were detected in the two duplicate samples, respectively (Additional file 2: Table **S1**). Genes with sgRNA abundance greater than 1000 and sgRNA frequency greater than 1 in two replicate samples were selected in the further analyses. Then the intersected 332 genes (Fig. 2a) were used for Kyoto encyclopedia of genes and genomes (KEGG) pathway and gene ontology (GO) analysis to determine the processes that mediated apatinib resistance. The top 10 enriched pathways by KEGG was showed in Fig. 2B, including cell cycle, ubiquitin mediated proteolysis, wnt signaling pathway, metabolic pathways, PI3K-Akt signaling pathway and so on (Fig. 2b). GO analysis showed the top 10 terms of molecular function, biological process and cellular component (Fig. 2c), which demonstrated their association with regulation of protein linear polyubiquitination, DNA-binding transcription repressor activity, etc. We focused on the wnt signaling and cell cycle pathways closely related to tumor growth and metastasis. Thus, a total of 7 candidate genes (MCM2, CCND3, ESPL1, PLK1, TCF7, PRICKLE1 and VANGL1) were selected, the heatmap and bar graph showed the sgRNAs abundance(Fig. 2d, left) and sgRNA frequency (Fig. 2d, right) respectively for the 7 selective genes. Scatter plot showed sgRNAs targeting the intersected 332 genes in two replicate samples and the ESPL1 have the highest sgRNA abundance among the 7 Candidate genes (Fig. 2e).

### Overexpression of candidate genes mediated apatinib resistance in vitro

**Fig. 3:**
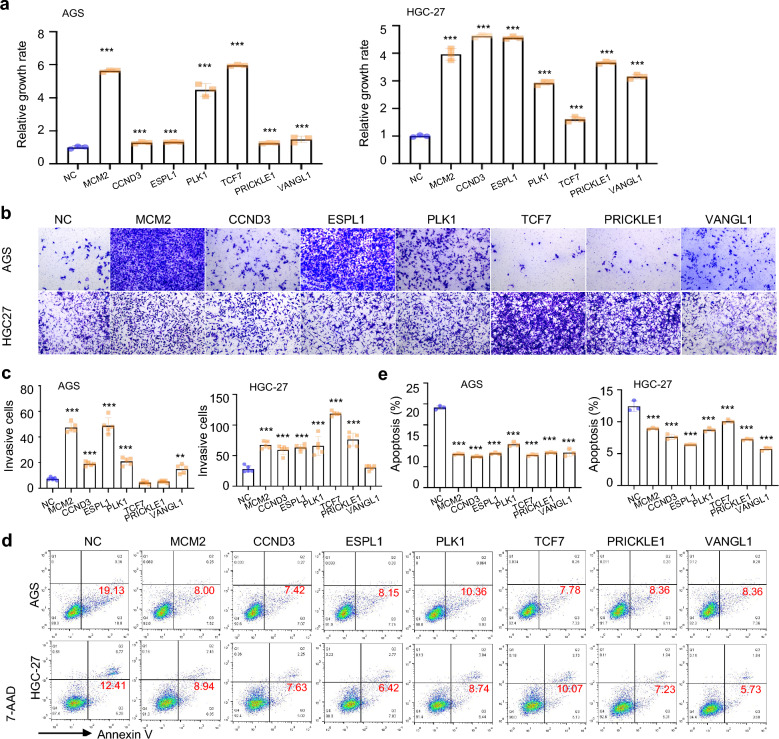
The overexpression of the candidate genes increases apatinib resistance in GC cell lines. **a** CCK8 assay was used to detect cell viability for AGS and HGC-27 cells with individual gene overexpression, relative growth rate was calculated based on the absorbance in NC; **b**, **c** Transwell assays clearly revealed the invasion cells of AGS and HGC-27 with individual gene overexpression at 24 h after treated with 10 μg/mL apatinib for 48 h; **d**, **e** Apoptosis assay was conducted to detect the effect of candidate gene overexpression on apatinib resistance in AGS and HGC-27 cells after treated with 10 μg/mL apatinib for 48 h. Statistical significance was determined by One-way ANOVA. Data were represented as means ± SD.**P < 0.01, ***P < *0.001* compared with the NC group

To validate whether the overexpression of the candidate genes mediate apatinib resistance in vitro. The sgRNA activation vectors (MCM2, CCND3, ESPL1, PLK1, TCF7, PRICKLE1 and VANGL1) were conducted and infected with GC cell lines AGS and HGC-27. The q-PCR assay was used to confirm the overexpression of these candidate genes (Additional file 1: Fig. **S1**). The sgRNA with higher overexpression efficiency were selected for further study. CCK8 assays showed that the overexpression of MCM2, CCND3, ESPL1, PLK1, TCF7, PRICKLE1 and VANGL1 could significantly promote GC cell proliferation after treatment with 10 μg/mL apatinib (*P* < 0.001) (Fig. 3a). Since apatinib is clinically used for the treatment of metastatic GC, the candidate genes were subjected to cell invasion experiment. We found the overexpression of MCM2, CCND3, ESPL1 and PLK1 remarkably promoted cell invasion after treatment with 10 μg/mL apatinib in AGS and HGC-27 cells (*P* < 0.001) (Fig. 3b, c). Moreover, flow apoptosis assay showed overexpression of the candidate genes could significantly inhibit cell apoptosis when treated with apatinib (*P* < 0.001) (Fig. 3d, e). The result supporting the view that gain function of the candidate genes in GC cells promoted apatinib resistance.

### Functional enrichment in the protein level of ESPL1

**Fig. 4:**
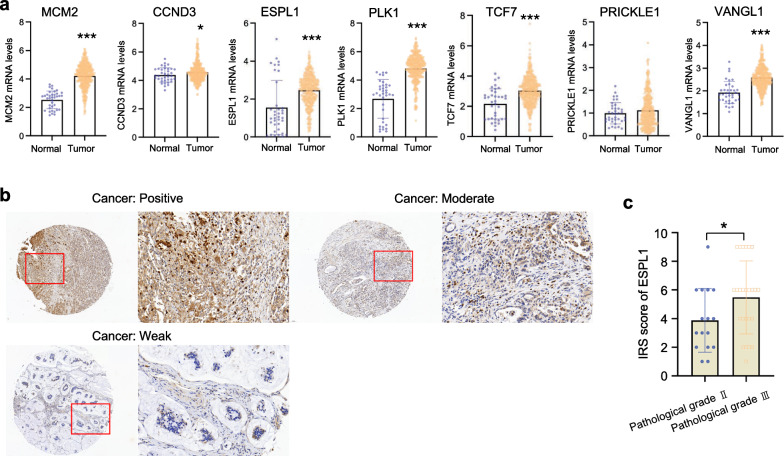
These candidate genes are valuable biomarkers in clinical GC samples. **a** The MCM2, CCND3, ESPL1, PLK1, TCF7 and VANGL1 mRNA expression was selectively up-regulated in GC from TCGA database; **b** ESPL1 protein showed positive, moderate or weak immunostaining in cell cytoplasmic of GC tissues from a tissue microarray; **c** ESPL1 protein level was significantly elevated in GC patients with pathological grade III (IRS, 5.48 ± 2.55, N = 25) compared with that in patients of pathological grade II (IRS, 3.88 ± 2.22, N = 16). Statistical significance was determined by t-test. Data were represented as means ± SD. *P < 0.05, **P < 0.01, ***P < 0.001, ****P < *0.0001* compared with the NC group

Then the RNA seq data from the cancer genome atlas (TCGA) were used to evaluate the expression of these candidate genes in the GC, which including 380 GC and 37 paracancerous samples. Of the identified hits, MCM2 (*P* < 0.001), CCND3 (*P* = 0.028), ESPL1 (*P* < 0.001), PLK1 (*P* < 0.001), TCF7 (*P* < 0.001) and VANGL1 (*P* < 0.001) mRNA expression was up-regulated in GC (Fig. 4a). As ESPL1 had the highest sgRNA abundance among the 7 candidate genes. Immunohistochemistry was used to detect ESPL1 protein level in 45 GC tissues. The result showed ESPL1 was positively expressed in GC cells and mainly located in the cytoplasm (Fig. 4b). Interestingly, we also found that ESPL1 protein level was significantly elevated in GC patients with pathological grade III (IRS, 5.48 ± 2.55) compared with that in patients of pathological grade II (IRS, 3.88 ± 2.22) (*P* < 0.05) (Fig. 4c). The result suggested that ESPL1 was closely associated with GC progression.

### Inhibition of ESPL1 enhanced the sensitivity of GC cells to apatinib

**Fig. 5:**
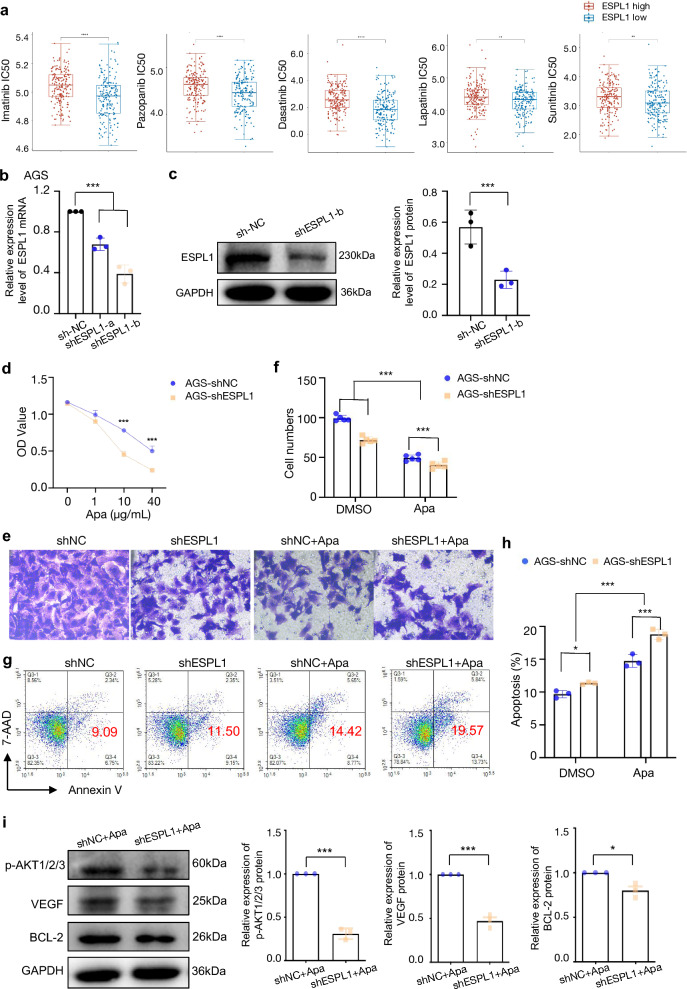
Inhibition of ESPL1 enhances the sensitivity of GC cells to apatinib. **a** Predicting the targeted therapeutics response for ESPL1 high expression group and low expression group of GC based on the Genomics of Drug Sensitivity in Cancer (GDSC); **b**, **c** The AGS cell lines were transfected with ESPL1 knockdown plasmid, q-PCR(b) and (c) was used to detect the knockdown efficiency of shESPL1; **d** CCK8 assay was used to detect cell proliferation after treated with 0, 1, 10 and 40 μg/mL apatinib of AGS-shNC and AGS-shESPL1 cells; **e**, **f** Transwell assays clearly revealed the invasion cells in AGS-shNC and AGS-shESPL1 group after treated with 10 μg/mL apatinib for 48 h; **g**, **h** Apoptosis assays showed the proportion of apoptotic cells in AGS-shNC and AGS-shESPL1 group after treated with 10 μg/mL apatinib for 48 h; **i** Western blots was conducted to detect p-AKT, VEGF and BCL-2 protein levels in AGS-shNC and AGS-shESPL1 group after treated with 10 μg/mL apatinib for 48 h(left), the Image J software was used for quantitative analysis(right). Statistical significance was determined by One-way ANOVA. Data were represented as means ± SD. *P < 0.05, **P < 0.01, ***P < *0.001* compared with the NC group

We downloaded the mRNA and clinical data of STADfrom the TCGA dataset. Then the therapeutic response was predicted by R package based on the GDSC dataset. As ESPL1 had the highest sgRNA abundance among the candidate targets. We focused on ESPL1 in the in subsequent research. The result showed that the IC_50_ of Imatinib, Pazopanib, Dasatinib, Lapatinib and Sunitinib was significantly higher in ESPL1 high expression group (*P* < 0.01) (Fig. 5a), which implying that ESPL1 reduced the sensitivity of these targeted drugs. Thus, we further explored whether inhibiting ESPL1 expression could improve sensitivity of GC cells to drug treatment. The AGS cells was transfected with ESPL1 shRNA plasmids (Table 1). Knockdown efficiency of ESPL1 was then demonstrated by q-PCR and western blots (Fig. 5b, c). Then, si-MDM2-c was chosen to interfere MDM2 protein level in the following studies. CCK8 assay was used to detect cell proliferation after treated with 0, 1, 10 and 40 μg/mL apatinib. The result showed that down-regulation of ESPL1 could significantly inhibit AGS cell proliferation when treated with different doses of apatinib (*P* < 0.001) (Fig. 5d). Transwell assays clearly revealed the invasion cells in AGS-shESPL1 group was significantly reduced compared to that of AGS-shNC group after treated with 10 μg/mL apatinib for 48 h (*P* < 0.001) (Fig. 5e-f). Apoptosis assays showed the proportion of apoptotic cells in AGS-shESPL1 group was significantly increased compared to that of AGS-shNC after treated with 10 μg/mL apatinib for 48 h (*P* < 0.001) (Fig. 5g-h). Western blots showed that inhibition of ESPL1 could significantly down-regulate the p-AKT1/2/3, VEGF and BCL-2 protein levels after treated with 10 μg/mL apatinib for 48 h (Fig. 5i) (*P* < 0.001, *P* < 0.001, *P* < 0.05, respectively). Taken together, these results demonstrated that inhibition of ESPL1 sensitized GC cells to apatinib treatment.

### The ESPL1 and MDM2 mRNAs were up-regulated in apatinib resistant GC cells

**Fig. 6:**
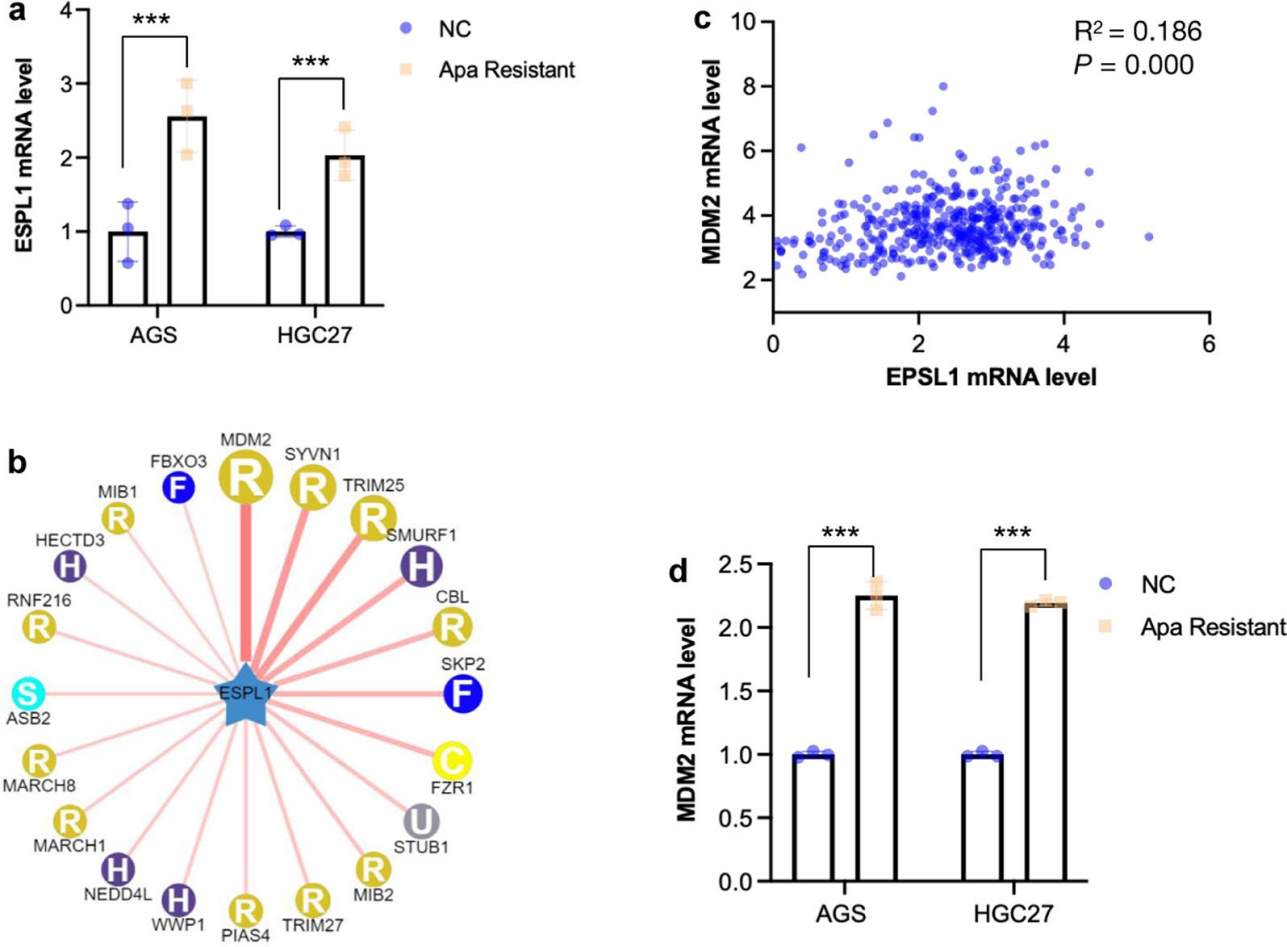
The ESPL1 and MDM2 mRNA level was up-regulated in apatinib resistant GC cells. **a** ESPL1 mRNA level in AGS and HGC27 cells was detected, the apatinib-resistant cells of GC cells were enriched by treating with 10 μg/mL apatinib for two weeks; **b** UbiBrowser was used to predict ubiquitin ligase of ESPL1. **c** Correlation analysis of ESPL1 and MDM2 by the mRNA data in TCGA; **d** MDM2 mRNA level in AGS and HGC27 cells was detected, the apatinib-resistant cells of GC cells were enriched by treating with 10 μg/mL apatinib for two weeks. Statistical significance was determined by One-way ANOVA and Pearson correlation analysis. Data were represented as means ± SD. ***P < *0.001* compared with the NC group

From the above studies, we found the overespression of ESPL1 lead to apatinib resistance. To determine if there is overexpression of ESPL1 in apatinib-resistant GC cells, the apatinib-resistant GC cells were enriched by treated with 10 μg/mL apatinib for two weeks and q-PCR was applied to detect the ESPL1 mRNA level. As shown in Fig. 6a, the ESPL1 mRNA level was significantly higher in apatinib-resistant GC cells than that in wild-type group (*P* < 0.001). To search for genes that might interact with ESPL1, UbiBrowser was used to predict ubiquitin ligase. Figure 6b showed MDM2 have highest score. Then the TCGA data was used to show the correlation between MDM2 and ESPL1. Correlation analysis showed that ESPL1 mRNA level is positively correlated with MDM2 (*P* = 0.000) (Fig. 6c). We then detected the MDM2 mRNA level in apatinib-resistant AGS cells and found that it was also significantly up-regulated (*P* < 0.001) (Fig. 6d). We deduced MDM2 might be closely associated with apatinib resistance.

### Inhibition of MDM2 could reverse ESPL1-mediated resistance to apatinib in GC cells

**Fig. 7:**
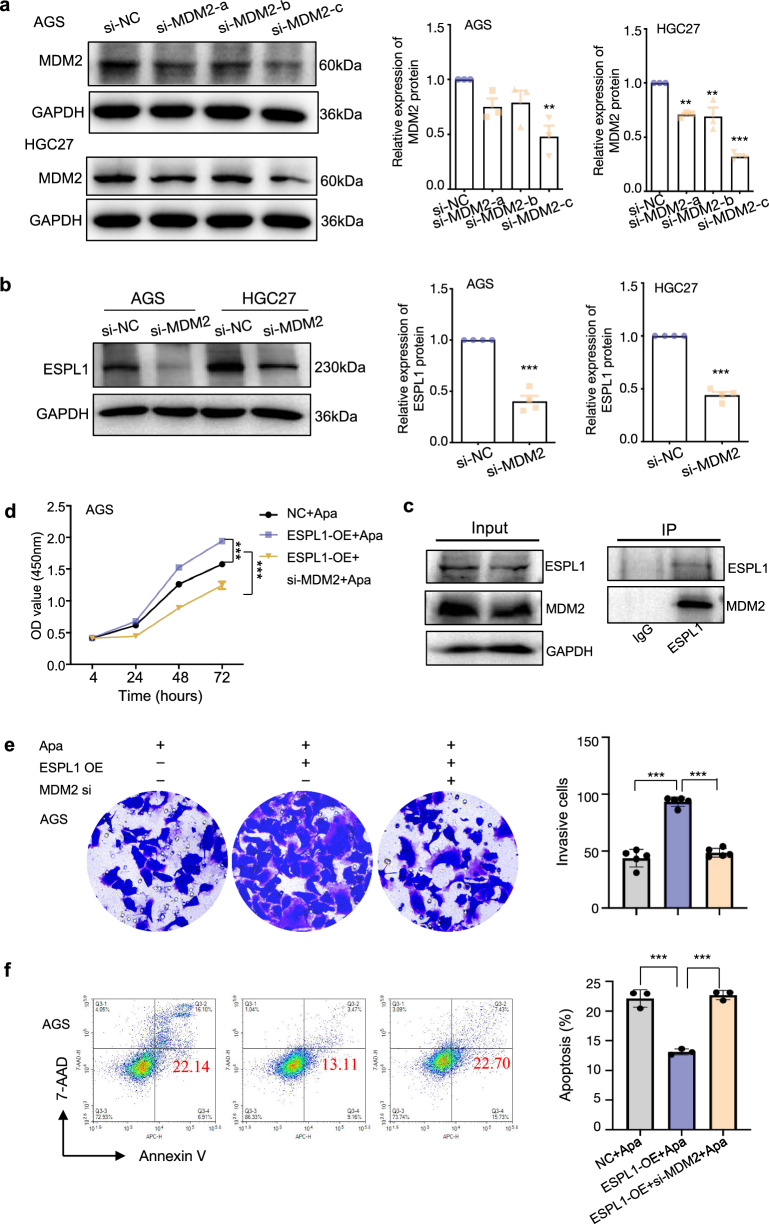
Inhibition of MDM2 rescued the sensitivity of ESPL1 overexpressing GC cells to apatinib. **a** The MDM2 siRNA was used to inhibit the expression of MDM2 and western blots was used to detect MDM2 protein levels in AGS and HGC27 cells (left), the Image J software was used for quantitative analysis (right); **b** Western blots was used to detect ESPL1 protein levels in MDM2 low-expression AGS and HGC27 cells(left), the Image J software was used for quantitative analysis (right); **c** Co-IP was used to detect the interaction between MDM2 and ESPL1 protein; **d** Time-course cell proliferation experiments of AGS-NC, AGS-ESPL1-OE, AGS-ESPL1-OE + siMDM2 cells under treatment of 10 μg/mL apatinib were detected by CCK8 at 4 h, 24 h, 48 h, 72 h; **e** Transwell assays clearly revealed the invasion cells in AGS-NC, AGS-ESPL1-OE, and AGS-ESPL1-OE + siMDM2 group after treated with 10 μg/mL apatinib for 48 h; **f** Apoptosis assays showed the proportion of apoptotic cells in AGS-NC, AGS-ESPL1-OE and AGS-ESPL1-OE + siMDM2 group after treated with 10 μg/mL apatinib for 48 h. Statistical significance was determined by One-way ANOVA. Data were represented as means ± SD. ***P < *0.001* compared with the NC group

The MDM2 protein is the primary negative regulatory factor of the p53 protein, which can ligate the p53 protein via its E3 ubiquitin ligase. Overexpression of MDM2 is associated with chemotherapeutic resistance through the p53-MDM2 loopdependent and p53-MDM2 loop-independent pathways in human malignancies [14]. We deduced high level of ESPL1 might lead to apatinib resistance through MDM2 pathways. The MDM2 siRNA (Table 2) was used to inhibit the expression of MDM2 and western blots was used to detect MDM2 protein levels in AGS and HGC27 cells, the results showed MDM2 siRNA can significantly interfere MDM2 protein level in the two GC cell lines (Fig. 7a) (*P* < 0.01). The ESPL1 protein level was down-regulated in si-MDM2 group, which was consistent with the expression level of MDM2 (Fig. 7b). Then Co-IP further confirmed the interaction of ESPL1 and MDM2 protein (Fig. 7c). Time-course cell proliferation experiments by CCK8 showed the OD value in ESPL1 overexpression plus siMDM2 group was remarkably reduced compared with that in ESPL1 overexpression group under treatment of 10 μg/mL apatinib (Fig. 7d) (*P* < 0.001). Transwell assays clearly revealed the significant reduction of invasion cells in ESPL1 overexpression plus siMDM2 group than that in ESPL1 overexpression group after treated with 10 μg/mL apatinib for 48 h (Fig. 7e) (*P* < 0.001). Apoptosis assays showed the proportion of apoptotic cells in ESPL1 overexpression plus siMDM2 group up-regulated significantly compared with that in ESPL1 overexpression group (Fig. 7f) (*P* < 0.001). These results indicated that inhibition of MDM2 could rescue the sensitivity of GC cells to apatinib and reverse ESPL1-mediated resistance.

## Discussion

Apatinib, a tyrosine kinase inhibitor (TKI), was the first anti-angiogenic agent approved for treatment of metastatic GC [3]. However, the emergence of resistance was inevitable. Previous studies proved FOXK2 induced apatinib resistance through VEGFA/VEGFR1 pathway in anaplastic thyroid cancer [15] and mutation of WRN induced apatinib resistance through activating the PI3K/AKT apoptosis-inhibiting pathway in non-small cell lung cancer [16]. Ye et al. found STING could sensitize head and neck squamous cell carcinoma cells to apatinib by decreasing ERBB2 expression and combination of STING agonist with apatinib synergistically ameliorated acquired apatinib resistance [17]. Therefore, exploring new and valuable off-target effect of apatinib directly against cancer cells is of great clinical significance.

Previous studies have reported the drug resistance mechanisms of multiple TKI drugs. Pan [18] et al. found that extracellular vesicle-packaged IGFL2-AS1 promotes Sunitinib resistance by regulating TP53INP2-triggered autophagy. Zeng [19] et al. reported the up-regulation of BCL6 following Imatinib treatment. Which lead to the tolerance of gastrointestinal stromal tumor cells by recruiting SIRT1 to the TP53 promoter to modulate histone acetylation and transcriptionally repress TP53 expression. Wei [20] et al. identified PHGDH as a critical driver for Sorafenib resistance in HCC by CRISPR/Cas9 library screening. In the study, the CRISPR activation library was used to screen the apatinib resistance targets. It was shown that the overexpression of MCM2, CCND3, ESPL1 and PLK1 remarkably promoted cell proliferation, invasion and inhibit cell apoptosis under the treatment of apatinib, which may mediate apatinib resistance in vitro. In addition, the expression of ESPL1mRNA was significantly higher in apatinib-resistant GC cells than that in wild-type GC cells, which established direct connection of ESPL1 with apatinib-resistance in GC.

ESPL1, a cysteine endopeptidase, was an oncogene and its overexpression was reported in a broad range of human tumors, including breast, brain, and prostate cancers [21]. Its activity could be controlled by direct binding of inhibitory proteins as well as posttranslational modification. Previous studied showed the activity of ESPL1 was negatively regulated by securin and Cdk1-cyclin B in vivo [6, 22]. The overactivated of ESPL1 linked to cancer and genome instability, thus it was an ideal target for drug discovery. Junryo R, et al. found ESPL1 was a downstream target of LAT3, which played an essential role in prostate cancer progression through the cellular uptake of essential amino acids [23]. Whether ESPL1 was involved in drug resistance has not yet been studied, while our study suggested ESPL1 was correlated with apatinib-resistance in GC.

In addition, we showed inhibition of ESPL1 sensitized GC cells to apatinib treatment. Additionally, western blots showed inhibition of ESPL1 could significantly down-regulate the p-AKT1/2/3, VEGF and BCL-2 protein levels after apatinib treatment, which demonstrated ESPL1 inhibition sensitized GC cells to apatinib might through suppressing cell proliferation and promoting apoptosis. The previous studies showed Sepin-1 was a potent non-competitive separase inhibitor that could inhibit cancer cell growth [24, 25]. Another well-known inhibitor securin could potently inhibit the separase catalytic activity by forming a tight complex [26]. In the near future, a combination therapy with the inhibitor Sepin-1 should be considered for apatinib-resistant patients with high ESPL1 expression, which requireed further verification.

We next performed correlation analysis with TCGA data to further explore the mechanism of ESPL1-mediated resistance to apatinib. According to our study, overexpression of ESPL1 might mediate apatinib resistance depending on the MDM2 pathway. Inhibition of MDM2 could reverse ESPL1-mediated resistance and rescue the sensitivity of GC cells to apatinib. MDM2 is a E3 ubiquitin protein ligase and is consided as a key negative regulator of p53. The amplification or overexpression of MDM2 is considered to be one of the main mechanisms of p53 degradation, and is associated with poor treatment response and clinical prognosis [27]. On one hand, MDM2 could prevent transcriptional activation of p53 and promote p53 degradation through ubiquitination. On the other hand, p53 could in turn stimulate transcription of MDM2 by binding to its promoter region [28]. Zhou, et al. discovered that MDM2 controls STAT5 stability in CD8 + T cells and was critical for effective antitumor immunity [29]. Some studies suggested that MDM2 increases drug resistance through inducing epithelial-mesenchymal transition independent of p53 [30]. Thus, whether the interaction between MDM2 and ESPL1 depends on the function of p53 is unclear. As targeting MDM2 and inhibiting its interaction with p53 was a promising strategy for cancer treatment [31]. The p53 mutant and wildtype GC cells should be used to explore the in-depth mechanism in the future.

In summary, our study was the first which employed unbiased whole genome CRISPR-library activation screening to systematically identify MCM2, CCND3, ESPL1 and PLK1 associated with apatinib resistance. In addition, we found inhibition of ESPL1 sensitized GC cells to apatinib treatment. Furthermore, the role of ESPL1 was dependent on MDM2 and combination of MDM2 siRNA with apatinib synergistically ameliorated acquired resistance induced by ESPL1 overexpression. Taken together, our findings indicated that targeting ESPL1 may be a promising strategy to overcome apatinib resistance in human GC.

### Supplementary Information

**Additional file 1: Fig. S1. **Quantitative real-time PCR analysis the high expression levels of the candidate genes. **a** MCM2, CCND3, ESPL1, PLK1, TCF7, PRICKLE1 and VANGL1 mRNA level in AGS cells was detected after transfected with corresponding sgRNA; **b** MCM2, CCND3, ESPL1, PLK1, TCF7, PRICKLE1 and VANGL1 mRNA level in HGC27 cells was detected after transfected with corresponding sgRNA. Statistical significance was determined by One-way ANOVA. Data were represented as means ± SD. *P < 0.05, **P < 0.01, ***P < 0.001 compared with the NC group.

**Additional file 2: Table S1.** The sgRNA sequencing result of the drug-resistant AGS cells.

## Data Availability

The data reported in this paper will be shared upon request to the lead corresponding author (gzhu17@gzhmu.edu.cn). Any additional information required to reanalyze the data reported in this paper is available from the lead contact upon request.
