# Supplementary figures and images for: Genome-wide CRISPR screen identifies ESPL1 limits the response of gastric cancer cells to apatinib
Source: Cancer Cell Int. 2024 Feb 24;24:83. doi: 10.1186/s12935-024-03233-4 (PMC10893712; doi:10.1186/s12935-024-03233-4)

**a** AGS

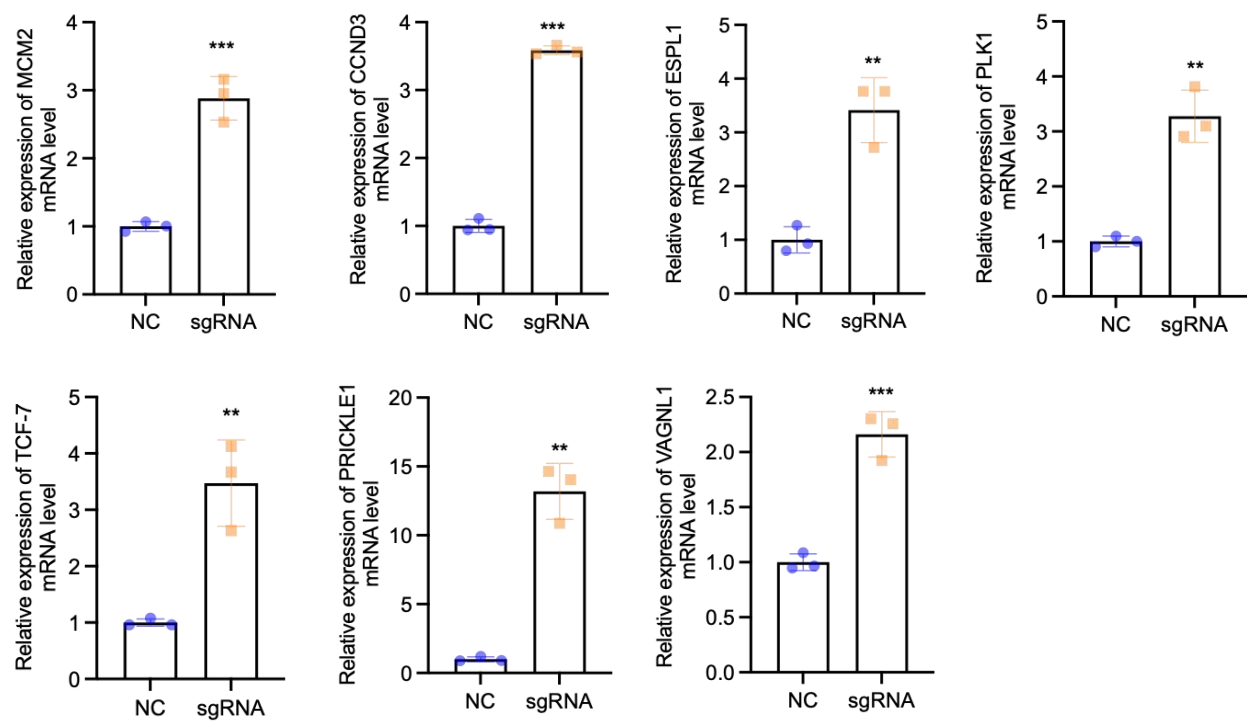

**b** HGC27

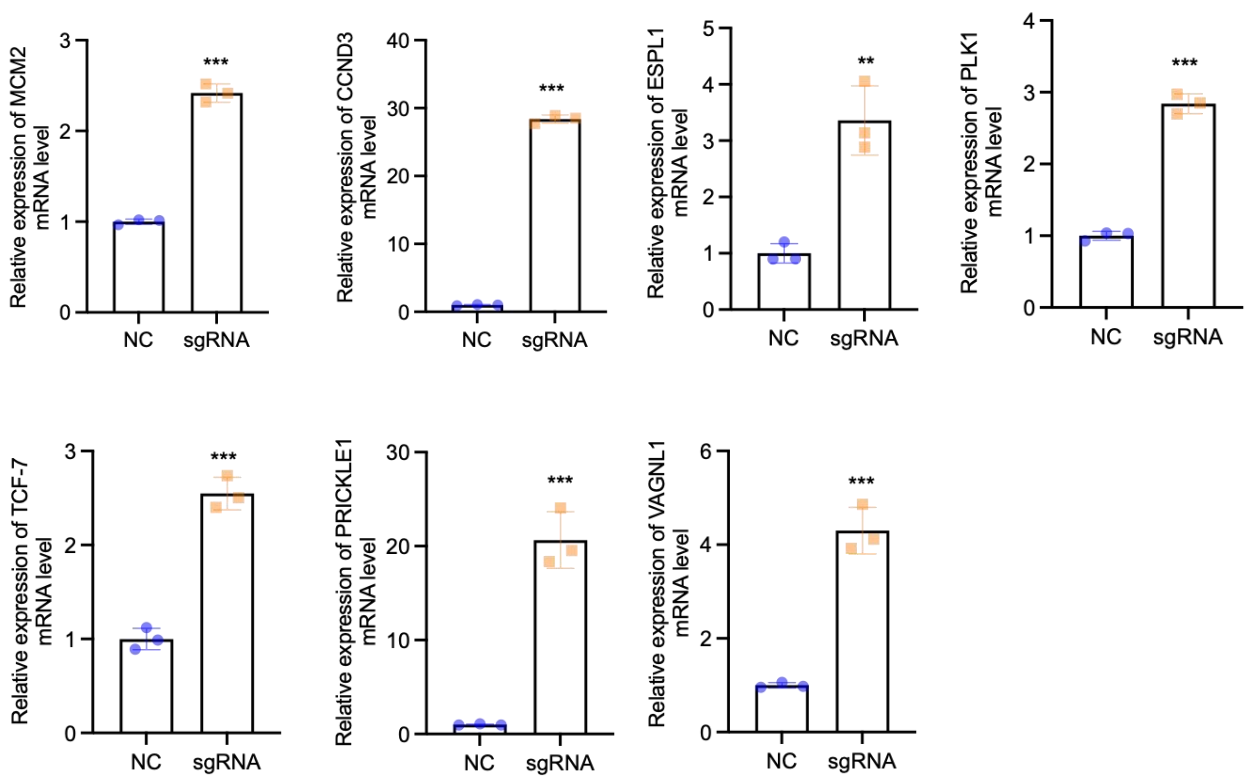

Supplement: Supplementary file 1 — Additional file 1: Fig. S1. Quantitative real-time PCR analysis the high expression levels of the candidate genes. a MCM2, CCND3, ESPL1, PLK1, TCF7, PRICKLE1 and VANGL1 mRNA level in AGS cells was detected after transfected with corresponding sgRNA; b MCM2, CCND3, ESPL1, PLK1, TCF7, PRICKLE1 and VANGL1 mRNA level in HGC27 cells was detected after transfected with corresponding sgRNA. Statistical significance was determined by One-way ANOVA. Data were represented as means ± SD. *P < 0.05, **P < 0.01, ***P < 0.001 compared with the NC group. [file 12935_2024_3233_MOESM1_ESM.pdf]
